# Infarct growth velocity predicts early neurological outcomes in single subcortical infarction

**DOI:** 10.1038/s41598-023-31727-0

**Published:** 2023-03-18

**Authors:** Ki-Woong Nam, Hyung-Min Kwon, Yong-Seok Lee

**Affiliations:** 1 Department of Neurology, Seoul Metropolitan Government-Seoul National University Boramae Medical Center, 20 Boramae-ro 5-gil, Dongjak-gu, Seoul, 07061 South Korea; 2 Seoul National University College of Medicine, Seoul, South Korea

**Keywords:** Stroke, Neurology, Neurological disorders

## Abstract

In single subcortical infarction (SSI), changes in lesion size are a major determinant of early neurological deterioration. We evaluated the association between END and infarct growth velocity (IGV) in patients with SSI. We included consecutive patients with SSI who underwent MRI within 24 h of symptom onset between 2010 and 2020. END was defined as an increase of ≥ 2 in the total National Institutes of Health Stroke Scale (NIHSS) score or ≥ 1 in the motor NIHSS score. IGV was calculated using the following formula: IGV (mL/h) = diffusion-weighted imaging volume (mL)/time to MRI (h). A total of 604 patients with SSI were evaluated. Multivariable logistic regression analysis showed that IGV remained significant after adjusting for confounders (aOR = 1.34, 95% CI 1.12–1.61). In a subgroup analysis based on the type of SSI, only patients with distal SSI showed an association between IGV and END (aOR = 1.64, 95% CI 1.24–2.16). In patients with proximal SSI, IGV did not show any statistical association with END. In conclusion, IGV was positively associated with END in patients with SSI. IGV should be interpreted differently in clinical settings depending on the location of the SSI lesion.

## Introduction

A single subcortical infarction (SSI) is an ischemic stroke of a single lesion found in the territory of perforating arteries^1^. Unlike patients with stroke due to other mechanisms, SSI patients usually present with mild neurological symptoms and have a favorable prognosis^2,3^. However, up to 43% of these patients experience a worsening of symptoms during acute period, and this development is referred to as early neurological deterioration (END)^4–6^. According to previous studies, a significant number of patients with SSI who experienced END events showed dependent functional outcomes at discharge^1,7,8^. Therefore, identifying the mechanism of END occurrence in these patients and preventing END are important. Researchers have identified the mechanisms and risk factors of END in SSI, and various clinical, laboratory, and radiological factors have been shown to be relevant^1,5,6,9–11^.

A perforating artery that develops SSI is very small and thin, and collateral flow is not well developed around the artery^1,12,13^. Therefore, this perforating artery forms a small ischemic penumbra and is less affected by external sources (e.g., cardioembolic source and proximal large artery disease). As a result, SSI has a relatively simple pathological mechanism; this is also true for END development^1,14^. The most important factors determining the prognosis and clinical course of SSI are the growth of the initial infarct lesion and the size of the final infarct^14,15^. For these factors, the number, location, and branching pattern of the perforating artery involved are important^13,15–17^. The presence or absence of plaque formation at the bifurcation between the parent artery and perforating artery also has an important effect^2,18–20^. If the morphology of the perforating artery can be observed using high-resolution MRI or if the growth velocity of lesions can be measured by performing a follow-up MRI, the occurrence of END and the prognosis could be predicted^2,18,21^. However, these technologies are not always available in clinical settings.

In this study, we evaluated the association between infarct growth velocity (IGV), which can be easily obtained using the initial infarct size and time from symptom onset, and END in patients with SSI. To identify the difference in the influence of IGV according to the location of the lesion or the pathophysiological mechanism, we also compared the association between IGV and END in proximal and distal SSI, respectively.

## Results

A total of 604 patients with SSI were analyzed (median age, 68 years; male sex, 56.8%; initial NIHSS score, 3). END occurred in 99 (16.4%) patients, and the median IGV value was 0.030 mL/h. Table 1 presents the details of the other baseline characteristics. In our data, IGV showed a positive correlation with diabetes, initial NIHSS score, fasting blood sugar level, white blood cell count, proximal SSI, DWI diameter, DWI volume, and END. IGV showed a negative correlation with time to MRI and posterior SSI (Supplementary Table 1).

**Table 1:** Baseline characteristics of the study population (n = 604).

Demographic and clinical factors	
Age, years [IQR]	68 [59–75]
Sex, male, n (%)	343 (56.8)
Time to MRI, h [SD]	10 [5–17]
Hypertension, n (%)	382 (63.2)
Diabetes, n (%)	191 (31.6)
Hyperlipidemia, n (%)	163 (27.0)
Current smoking, n (%)	192 (31.8)
Stroke history, n (%)	75 (12.4)
Initial NIHSS score, [SD]	3 [1–4]
Laboratory factors	
Fasting blood sugar, mg/dL [SD]	97 [88–116]
Hemoglobin A1c, % [SD]	5.9 [5.5–6.8]
Total cholesterol, mg/dL [SD]	184 [156–213]
White blood cell counts, × 10^3^/μL [SD]	6.98 [5.60–8.55]
High-sensitivity CRP, mg/dL [SD]	0.11 [0.05–0.31]
Radiological factors	
Involved vascular territory, n (%)	
Anterior SSI	384 (63.6)
Posterior SSI	220 (36.4)
SSI type, n (%)	
Proximal SSI	181 (30.0)
Distal SSI	423 (70.0)
DWI diameter, mm [SD]	10 [7–13]
DWI volume, mL [SD]	0.31 [0.15–0.63]
Infarct growth velocity, mL/h [SD]	0.030 [0.014–0.082]
Outcome factors	
Early neurological deterioration, n (%)	99 (16.4)
Unfavorable outcome (mRS ≥ 3)	134 (22.2)

*MRI* magnetic resonance imaging, *NIHSS* National Institutes of Health Stroke Scale, *CRP* C-reactive protein, *SSI* single subcortical infarction, *DWI* diffusion-weighted imaging, *mRS* modified Rankin Scale.

In the univariate analysis, END was significantly associated with age, sex, initial NIHSS score, SSI type, DWI diameter, DWI volume, and IGV (Table 2). In the multivariable logistic regression analysis, IGV remained significant after adjusting for confounders (aOR = 1.34, 95% CI 1.12–1.61). Age (aOR = 1.04, 95% CI 1.01–1.06) and proximal SSI (aOR = 1.96, 95% CI 1.20–3.22) were also positively associated with END, independent of IGV. Based on the receiver operating characteristic curve, the most appropriate cut-off point for IGV for END prediction in our study population was 0.038 mL/h (Supplementary Fig. 1). When the multivariable analysis was performed on the basis of this cut-off point, “ICGV ≥ 0.038 mL/h” showed a close statistical association with END (aOR = 2.13, 95% CI 1.30–3.49; Table 3).

**Table 2:** Baseline characteristics of groups with and without early neurological deterioration.

	Non-END (n = 505)	END (n = 99)	*P*-value
Age, years [IQR]	67 [58–74]	73 [65–80]	< 0.001
Sex, male (%)	296 (58.6)	47 (47.5)	0.041
Time to MRI, h [IQR]	10.0 [5.0–18.5]	9.0 [5.0–15.5]	0.118
Hypertension, n (%)	314 (62.2)	68 (68.7)	0.219
Diabetes, n (%)	158 (31.3)	33 (33.3)	0.689
Hyperlipidemia, n (%)	140 (27.7)	23 (23.2)	0.357
Current smoking, n (%)	168 (33.3)	24 (24.2)	0.078
Stroke history, n (%)	65 (12.9)	10 (10.1)	0.441
Initial NIHSS score [IQR]	2 [1–4]	3 [2–5]	< 0.001
Fasting blood sugar, mg/dL [IQR]	97 [88–115]	101 [88–122]	0.487
Hemoglobin A1c, % [IQR]	5.9 [5.5–6.8]	6.0 [5.6–7.0]	0.210
Total cholesterol, mg/dL [IQR]	182 [156–212]	192 [158–216]	0.376
White blood cell counts, × 10^3^/μL [IQR]	6.98 [5.65–8.52]	6.85 [5.58–8.61]	0.869
hs-CRP, mg/dL [IQR]	0.11 [0.05–0.32]	0.13 [0.07–0.28]	0.239
Involved vascular territory, n (%)			0.166
Anterior SSI	315 (62.4)	69 (69.7)	
Posterior SSI	190 (37.6)	30 (30.3)	
SSI type, n (%)			< 0.001
Proximal SSI	370 (73.3)	53 (53.5)	
Distal SSI	135 (26.7)	46 (46.5)	
DWI diameter, mm [IQR]	9 [6–13]	12 [8–18]	< 0.001
DWI volume, mL [IQR]	0.28 [0.14–0.53]	0.52 [0.28–1.15]	< 0.001
Infarct growth velocity, mL/h [IQR]	0.027 [0.013–0.060]	0.078 [0.027–0.153]	< 0.001

*END* early neurological deterioration, *MRI* magnetic resonance imaging, *NIHSS* National Institutes of Health Stroke Scale, *hs-CRP* high-sensitivity C-reactive protein, *SSI* single subcortical infarction, *DWI* diffusion-weighted imaging.

**Table 3:** Multivariable logistic regression analysis of possible predictors of early neurological deterioration.

	Crude OR (95% CI)	*P-*value	Adjusted OR (95% CI)	*P-*value
Model 1 (continuous)				
Age	1.05 [1.03–1.07]	< 0.001	1.04 [1.01–1.06]	0.003
Sex	0.64 [0.41–0.98]	0.042	0.76 [0.45–1.30]	0.321
Initial NIHSS score	1.77 [1.25–2.51]	0.001	1.29 [0.88–1.87]	0.191
Current smoking	0.64 [0.39–1.05]	0.080	0.95 [0.50–1.82]	0.880
Proximal SSI	2.38 [1.53–3.70]	< 0.001	1.96 [1.20–3.22]	0.008
IGV*	1.47 [1.26–1.71]	< 0.001	1.34 [1.12–1.61]	0.001
Model 2 (categorical)				
Age	1.05 [1.03–1.07]	< 0.001	1.03 [1.01–1.06]	0.003
Sex	0.64 [0.41–0.98]	0.042	0.74 [0.43–1.25]	0.254
Initial NIHSS score	1.77 [1.25–2.51]	0.001	1.35 [0.93–1.96]	0.117
Current smoking	0.64 [0.39–1.05]	0.080	0.90 [0.47–1.73]	0.761
Proximal SSI	2.38 [1.53–3.70]	< 0.001	2.09 [1.28–3.40]	0.003
IGV ≥ 0.038 mL/h	2.51 [1.61–3.91]	< 0.001	2.13 [1.30–3.49]	0.003

*NIHSS* National Institutes of Health Stroke Scale, *SSI* single subcortical infarction, *IGV* infarct growth velocity.

*These variables were transformed into a log scale.

**Figure 1:**
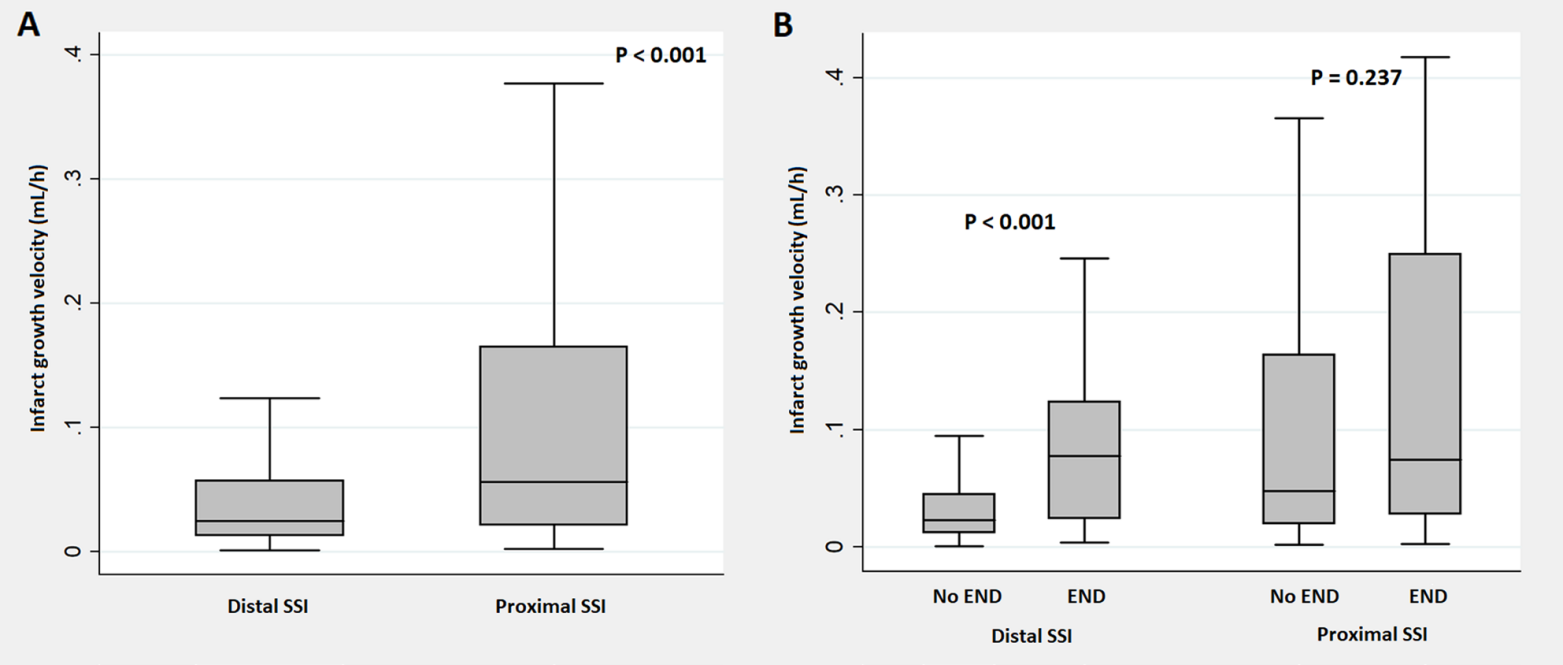
Comparison of infarct growth velocity in patients with distal and proximal single subcortical infarction according to the presence of early neurological deterioration. *IGV* infarct growth velocity, *SSI* single subcortical infarction, *END* early neurological deterioration. IGV values were higher in proximal SSI than in distal SSI (*P* < 0.001). Significant differences in IGV values ​according to END were found only in distal SSI (*P* < 0.001). No statistical significance was found in proximal SSI (*P* = 0.237).

In our data, IGV values were higher in proximal SSI than in distal SSI (*P* < 0.001) and a significant difference in IGV values according to the presence or absence of END was found only in distal SSI (Fig. 1). Thus, the biological interaction between IGV and the type of SSI was examined, and significant statistical values were obtained (Supplementary Table 2). In a subgroup univariate and multivariable analyses based on the type of SSI, patients with distal SSI showed a clear association between IGV and END (IGV − [continuous variable]: aOR = 1.64, 95% CI 1.24–2.16; IGV ≥ 0.038 mL/h − [categorical variable]: aOR = 3.49, 95% CI 1.78–6.84). In patients with proximal SSI, IGV did not show any statistical association with END (Supplementary Table 3 and Table 4).

**Table 4:** Relationship between infarct growth velocity and early neurological deterioration according to the location of single subcortical infarction lesion.

	Crude OR (95% CI)	*P-*value	Adjusted OR^†^ (95% CI)	*P-*value
Distal SSI				
IGV* (continuous)	1.79 [1.41–2.28]	< 0.001	1.64 [1.24–2.16]	< 0.001
IGV ≥ 0.038 (categorical)	3.57 [1.96–6.48]	< 0.001	3.49 [1.78–6.84]	< 0.001
Proximal SSI				
IGV* (continuous)	1.09 [0.88–1.36]	0.417	1.09 [0.86–1.38]	0.489
IGV ≥ 0.038 (categorical)	1.07 [0.54–2.12]	0.848	0.96 [0.46–2.03]	0.915

*SSI* single subcortical infarction, *IGV* infarct growth velocity.

*These variables were transformed into a log scale.

^†^These adjusted OR values were obtained by adjusting the variables with *P* < 0.10 as confounders in a univariate analysis performed respectively for each group of patients with SSI of different locations.

The unfavorable discharge outcome based on an mRS score ≥ 3 showed a close correlation with END (*P* < 0.001). In addition, DWI volume, IGV, and initial NIHSS score were also associated with unfavorable outcomes. Time to MRI had no effect on discharge outcome (Supplementary Table 4).

## Discussion

In this study, we found that IGV was positively associated with END in patients with SSI. This association was prominent in patients with distal SSI, but was not statistically significant in patients with proximal SSI. Therefore, IGV needs to be interpreted differently in clinical settings depending on the location of the SSI lesion.

Given that END in patients with SSI is mainly caused by an increase in the size of the initial lesion, the final infarct size and the anatomical positional relationship between the lesion and corticospinal tract determine the occurrence of END^14,22^. From this point of view, the close relationship between IGV and END can be interpreted as follows. First, a high IGV means a large final infarct size. To produce an infarct lesion in a large area within a short period, a penetrating artery branching to a broad area must be invaded, or several penetrating arteries must be involved at the same time^15,21,23,24^. This means that the size of the final infarct will be large, thus increasing the chance of interrupting the corticospinal tract. Second, IGV can also reveal a vulnerable brain environment. Pathological conditions such as cerebral small vessel disease, which is often found with SSI, can create a hostile brain environment susceptible to ischemic insults^25,26^. In such an environment, faster and larger lesions can be created with the same ischemic insult. In particular, the territory of the penetrating arteries, which has difficulty receiving assistance from collateral flow, is thought to be more influenced by such a hostile environment. Although we did not confirm it in our data, patients with high IGV had a more vulnerable brain environment than patients with low IGV; therefore, patients with high IGV may experience a larger final infarct even with the same ischemic insult. Last, patients who visited a hospital earlier could have a greater chance of having their END events discovered during hospitalization. However, the median time from symptom onset to MRI scan in our study population was relatively fast at 10 [5–17] h. Statistical significance was lost when we introduced the variable “time to MRI” instead of IGV in the multivariable analysis. Therefore, the influence of not observing the END event due to the late visit time does not seem to be significant.

Interestingly, the close association between IGV and END appeared only in distal SSI and lost its influence in proximal SSI. IGV and the type of SSI showed distinct biological interactions. This may be due to the difference in lesion size between proximal and distal SSI. Proximal SSI is known to have a larger lesion size than distal SSI, and this fact was confirmed by the significant difference in IGV values in our data^19,20,27,28^. Therefore, a slight change in IGV value may have a greater significance in distal SSI with a smaller lesion volume than in proximal SSI. Furthermore, proximal SSI occurs when the perforating artery/arteriole is occluded at the orifice level^1,20,29^. As mentioned earlier, considering that collateral flow is not developed well in these blood vessels, the occlusion of the orifice eventually causes blood supply failure to the distal area. Even if the initial lesion and IGV value are small, the proximal SSI has a high possibility of progression; therefore, the predictive power of IGV may be relatively poor. However, our results can also be interpreted as follows by considering the anatomical locations of the major structures. Above all, the trajectory of the corticospinal tract should be considered. In the posterior circulation, the corticospinal tract runs along the ventral surface of the brainstem^1,2^. Therefore, a proximal SSI that originates from the basilar-perforating artery can easily invade the corticospinal tract even if its size is small. In the case of anterior circulation, the corticospinal tract passes through the posterior superior segment of the striatocapsular area, and the vessels responsible for this are the lateral lenticulostriate artery and the anterior choroidal artery^1,6,12,22,30^. At this time, given that an SSI occurring in the anterior choroidal artery is mostly classified as a proximal type, it is likely to affect the corticospinal tract that travels nearby regardless of the lesion size^15,22^. On the other hand, in the case of distal SSI, given that lesions are formed somewhat distant from the corticospinal tract in both the anterior and posterior circulation, whether the corticospinal tract can be involved depends on lesion size. Therefore, the IGV value may have a more important effect on END generation for distal SSI than for proximal SSI.

The application of IGV may be questionable for someone because it is already a well-known fact that the initial lesion volume determines the early neurological outcome in patients with SSI. However, with the addition of the variable of time, IGV obtains a more complex meaning. If even a small lesion shows a high IGV value, it means that the lesion was formed in a short amount of time. These patients may have vulnerable brain tissue or problems in large or multiple perforating arteries. IGV can indicate that the lesion is likely to expand during the acute phase and that END is likely to occur. On the other hand, if the IGV value is low even if the lesion is large, it means that a significant amount of time has passed. After all, in these patients, the lesion has already entered a stable state, or the brain tissue is resistant to ischemic insults to that extent, thus indicating that there will be no changes in the size of the additional lesion. In an environment where it is difficult to take several follow-up MRIs, we expect that this information can be used in a useful and economical manner to predict the prognosis of patients with distal SSI. However, our results need to be verified by further studies in several study populations.

There are several caveats to consider when interpreting the findings of this study. First, our study is a retrospective cross-sectional study. Owing to the nature of the cross-sectional analysis, we can establish an association between IGV and END but not a causal relationship. Second, some patients with SSI experience capsular warning syndrome during the acute period^31,32^. Therefore, it is possible that some of the events we measured as END were transient capsular warning syndromes. However, in our results, END was statistically and distinctly related to the discharge outcome; therefore, the influence of capsular warning syndrome was not significant enough to change our main results. Third, we used a relatively sensitive definition of END, which may have overestimated the prevalence of END^33^. However, this definition was also widely used in other studies and showed a statistically strong correlation with our discharge functional outcome^34,35^. Therefore, there seems to be no major problem in using it as an outcome variable. Fourth, we calculated IGV under the assumption that the lesion would linearly increase in size over time. However, the actual change in the lesion size may be slightly different from this assumption. Last, the possibility of selection bias in the process of selecting the study population should also be considered. To focus on the mechanisms of END related to the perforating artery, we excluded patients with other potent external embolic sources that could affect stroke development and progression. This simplified the interpretation of the pathological mechanisms, but the problem of selection bias must be considered to some extent.

We demonstrated that IGV was associated with END in patients with SSI. Our findings suggested the possibility of classifying the high-risk group of END events by obtaining IGV in a simple manner at the same time when DWI images were taken. Given that END was closely related to discharge outcome, IGV seems to have clinical significance for patients with SSI, particularly distal SSI. However, our findings should be validated by future prospective studies.

## Methods

### Study population

We included patients with SSI who underwent MRI within 24 h of symptom onset between January 2010 and December 2020 from a consecutive ischemic stroke registry at a large stroke center in South Korea (Seoul Metropolitan Government-Seoul National University Boramae Medical Center). SSI was defined as a single infarct located in the territory of the perforating arteries. In accordance with our center’s protocol, all patients with SSI underwent extensive etiological evaluations, including brain MRI; MRA; echocardiography; and laboratory examinations, such as those for patients with other types of ischemic stroke^1^. Patients who met the following exclusion criteria were excluded from the analysis: (1) patients without brain MRI and MRA data, (2) patients who received thrombolytic therapy, and (3) patients who have a high risk of having other stroke mechanisms (e.g., cardioembolic sources or intracranial/extracranial atherosclerosis with more than 50% stenosis). In total, 604 patients with SSI were included in the final analysis.

This retrospective study was approved by the IRB of Seoul Metropolitan Government-Seoul National University Boramae Medical Center (number: 20-2020-8). The IRB of Seoul Metropolitan Government-Seoul National University Boramae Medical Center waived the requirement for written informed consent because of the retrospective nature of the study and because the study used only de-identified anonymized patient information. All experiments were conducted according to the Declaration of Helsinki and all relevant guidelines and regulations.

### Clinical assessment

We evaluated baseline demographic, clinical, and vascular risk factors, including age, sex, time to MRI, hypertension, diabetes, hyperlipidemia, current smoking, previous stroke history, and initial stroke severity^1^. Time to MRI was defined as the time taken from when a patient first noticed symptoms until the MRI was taken at our center. The initial stroke severity was rated using the National Institutes of Health Stroke Scale (NIHSS) score on a daily basis from admission to discharge date by well-trained neurologists who were not involved in this study. Laboratory examinations were performed within the first 24 h after admission and included glucose profiles, lipid profiles, and inflammatory markers.

As the main outcome of this study, we defined END as an increase of ≥ 2 points in the total NIHSS score or ≥ 1 in the motor NIHSS score within the first 72 h of admission^1^. To comprehensively understand the effects of IGV on early prognosis in patients with SSI, we evaluated the discharge outcome as an additional outcome variable by using the modified Rankin Scale (mRS) score. On the bases of the mRS scores at the time of discharge, we classified patients with ≥ 3 points into the unfavorable outcome group and the rest of the patients into the favorable outcome group.

### Radiological assessment

**Figure 2:**
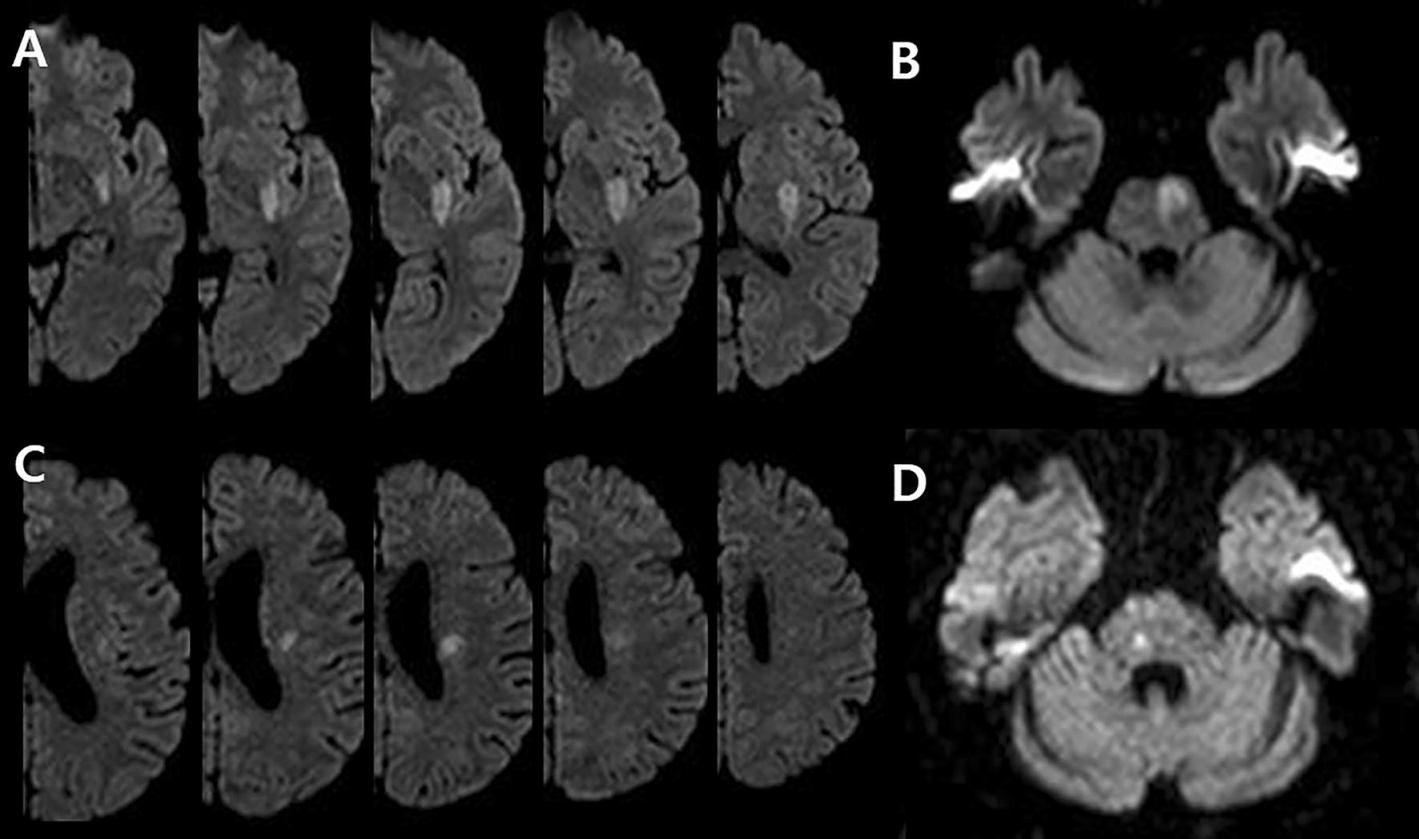
Representative examples of distal and proximal single subcortical infarction in the anterior and posterior circulation. *SSI* single subcortical infarction. (**A**) Anterior proximal SSI, (**B**) posterior proximal SSI, (**C**) anterior distal SSI, and (**D**) posterior distal SSI.

All patients underwent brain MRI and MRA within 24 h of admission via a 3.0-T magnetic resonance scanner (Achieva 3.0T; Philips, Eindhoven, the Netherlands). SSI was defined as a single lesion that did not involve the cerebral cortex but invaded the perforating artery in the middle cerebral artery, posterior cerebral artery, or basilar artery (e.g., striatocapsular, thalamic, or brainstem area)^1^. According to the location of the SSI lesion, we divided the involved vascular territory and the type of SSI^1^. The involved vascular territory was simply divided into anterior SSI and posterior SSI according to circulation. The type of SSI was divided into two types: proximal and distal. Proximal SSI was defined as an infarction adjacent to the parent artery and extended toward the basal surface of the parent artery^1^. Distal SSI was defined as an infarction that was located only in the distal area and was not in contact with the parent artery (Fig. 2)^1^. We measured the diameter (mm) and volume (mL) of the diffusion-weighted imaging (DWI) lesion, which are characteristics of SSI lesions. DWI volumes were measured quantitatively using Medical Imaging Processing, Analysis, and Visualization software (MIPAV version 11.0.0; National Institutes of Health, Bethesda, MD, USA). Volume measurement was performed using computer-assisted semi-automated technology on data obtained from converted files that are in DICOM format, similar to that in previous studies^36^.

**Figure 3:**
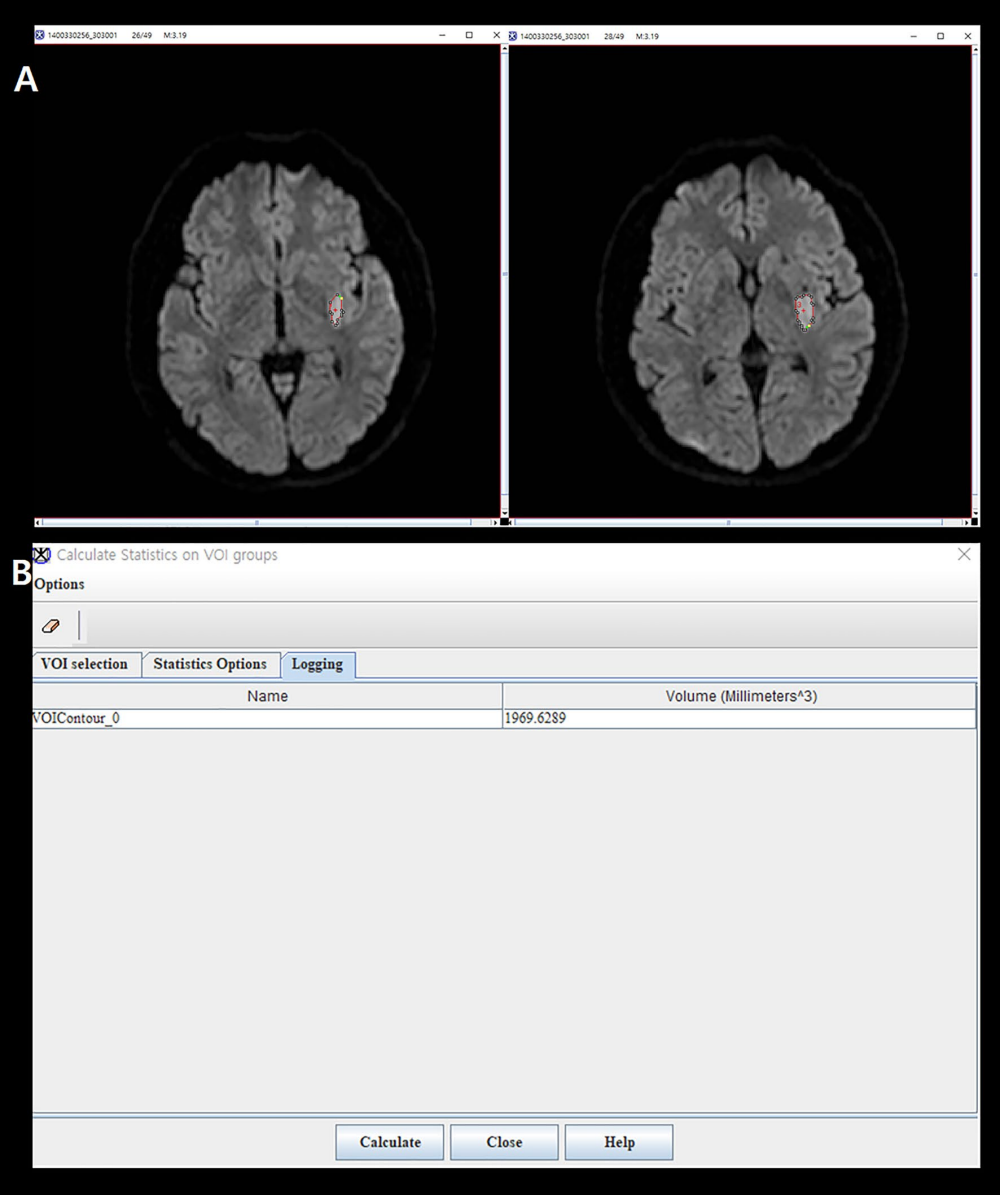
A representative example of how to calculate the infarct growth velocity. We quantitatively measured initial infarct volume using MIPAV version 11.0.0. program. The infarct lesions of each slice were delineated in a semi-automated way. After that, the program automatically calculated the volume by summing the lesion area of each slice. The initial infarct volume measured in this way was 1.97 mL, and MRI was conducted in this patient 1 h after symptom onset. Therefore, according to the formula, it is calculated that this patient had an IGV value of 1.97 mL/h.

SSI is a disease that exists as a single lesion from symptom onset to hospital discharge. Also, since the actual growth velocity of lesions is unpredictably complex, we simply assumed that they follow a linear trend. Based on this background, we hypothesized that approximate IGV could be measured by dividing the size of the lesion on the initial MRI by the time from symptom onset to image acquisition. That is, in simple terms, IGV can be calculated with the following formula: IGV (mL/h) = DWI volume (mL)/time to MRI (h) (Fig. 3). All radiological parameters were rated by two neurologists (K.-W.N. and H.-M.K.), and disagreements were resolved via discussions with a third rater (Y.-S.L.).

### Statistical analysis

All statistical analyses were performed using SPSS version 23.0 (IBM Corp. Chicago, IL, USA). Continuous variables following normal and non-normal distributions are presented as mean ± SD and median + interquartile range, respectively. Univariate analyses for identifying possible predictors for END were conducted using Student’s *t*-test or the Mann–Whitney *U*-test for continuous variables and the chi-squared test for categorical variables. Variables with *P* < 0.10 from the results of univariate analyses were considered confounders in the multivariable logistic regression analysis. Considering the close interaction with IGV, the time to MRI, DWI diameter, and DWI volume were not included in the multivariable analysis.

Given that the volume of the SSI lesion may vary depending on its location, the IGV may also vary. By taking this into account, we tried to examine whether the type of SSI or the vascular territory involved affects the association between IGV and END via biological interactions. Thus, we created a new interaction variable and analyzed it by using multivariable logistic regression analysis. All variables with *P* < 0.05 were considered significant in this study.

## Supplementary Information

Supplementary Information 1.

Supplementary Information 2.

## Data Availability

All data generated or analyzed during this study are included in this published article [and its supplementary information files].

## References

[1] Nam K-W, Kwon H-M, Lee Y-S (2021). Different predictive factors for early neurological deterioration based on the location of single subcortical infarction: Early prognosis in single subcortical infarction. Stroke.

[2] Kim JS, Yoon Y (2013). Single subcortical infarction associated with parental arterial disease: Important yet neglected sub-type of atherothrombotic stroke. Int. J. Stroke.

[3] Duan Z (2015). Acute diffusion-weighted imaging lesion patterns predict progressive small subcortical infarct in the perforator territory of the middle cerebral artery. Int. J. Stroke.

[4] Jiang J (2019). Total MRI burden of cerebral vessel disease correlates with the progression in patients with acute single small subcortical strokes. Brain Behav..

[5] Jeong H-G, Kim BJ, Yang MH, Han M-K, Bae H-J (2015). Neuroimaging markers for early neurologic deterioration in single small subcortical infarction. Stroke.

[6] Kim JP (2015). Diffusion-perfusion mismatch in single subcortical infarction: A predictor of early neurological deterioration and poor functional outcome. Eur. Neurol..

[7] Helleberg BH, Ellekjaer H, Indredavik B (2016). Outcomes after early neurological deterioration and transitory deterioration in acute ischemic stroke patients. Cerebrovasc. Dis..

[8] Nam K-W (2021). Triglyceride-glucose index is associated with early neurological deterioration in single subcortical infarction: Early prognosis in single subcortical infarctions. Int. J. Stroke.

[9] Ryu J-C (2022). Blood pressure variability and early neurological deterioration according to the chronic kidney disease risk categories in minor ischemic stroke patients. PLoS One.

[10] Castellanos M (2002). Inflammation-mediated damage in progressing lacunar infarctions: A potential therapeutic target. Stroke.

[11] Serena J (2001). Neurological deterioration in acute lacunar infarctions: The role of excitatory and inhibitory neurotransmitters. Stroke.

[12] Kim SK (2008). Prediction of progressive motor deficits in patients with deep subcortical infarction. Cerebrovasc. Dis..

[13] Hallevi H (2012). Intracranial atherosclerosis is associated with progression of neurological deficit in subcortical stroke. Cerebrovasc. Dis..

[14] Cho K-H, Kang D-W, Kwon SU, Kim JS (2009). Lesion volume increase is related to neurologic progression in patients with subcortical infarction. J. Neurol. Sci..

[15] Kim BJ, Lee DH, Kang D-W, Kwon SU, Kim JS (2014). Branching patterns determine the size of single subcortical infarctions. Stroke.

[16] Takase K-I (2011). Initial MRI findings predict progressive lacunar infarction in the territory of the lenticulostriate artery. Eur. Neurol..

[17] Nah H-W, Kang D-W, Kwon SU, Kim JS (2010). Diversity of single small subcortical infarctions according to infarct location and parent artery disease: Analysis of indicators for small vessel disease and atherosclerosis. Stroke.

[18] Kim BJ (2015). The shape of middle cerebral artery and plaque location: High-resolution MRI finding. Int. J. Stroke.

[19] Wen L, Feng J, Zheng D (2013). Heterogeneity of single small subcortical infarction can be reflected in lesion location. Neurol. Sci..

[20] Jiang S (2020). Plaque distribution correlates with morphology of lenticulostriate arteries in single subcortical infarctions. Stroke.

[21] Yoon Y, Lee DH, Kang D-W, Kwon SU, Kim JS (2013). Single subcortical infarction and atherosclerotic plaques in the middle cerebral artery: High-resolution magnetic resonance imaging findings. Stroke.

[22] Terasawa Y (2008). Neurological deterioration in small vessel disease may be associated with increase of infarct volume. J. Neurol. Sci..

[23] Gao Y (2017). Vascular lesion thickness in the lenticulostriate artery region serves as a biomarker for early neurological deterioration. Curr. Neurovasc. Res..

[24] Shen M (2018). Middle cerebral artery atherosclerosis and deep subcortical infarction: A 3T magnetic resonance vessel wall imaging study. J. Stroke Cerebrovasc. Dis..

[25] Wardlaw JM (2013). Neuroimaging standards for research into small vessel disease and its contribution to ageing and neurodegeneration. Lancet Neurol..

[26] Feng C (2014). Leukoaraiosis correlates with the neurologic deterioration after small subcortical infarction. J. Stroke Cerebrovasc. Dis..

[27] Zhang C (2014). Distal single subcortical infarction had a better clinical outcome compared with proximal single subcortical infarction. Stroke.

[28] Gao Y (2016). Pathogenic heterogeneity of distal single small subcortical lenticulostriate infarctions based on lesion size. J. Stroke Cerebrovasc. Dis..

[29] Yan Y (2023). Lenticulostriate artery length and middle cerebral artery plaque as predictors of early neurological deterioration in single subcortical infarction. Int. J. Stroke.

[30] Yang X (2014). The infarct location predicts the outcome of single small subcortical infarction in the territory of the middle cerebral artery. J. Stroke Cerebrovasc. Dis..

[31] Donnan GA, Omalley H, Quang L, Hurley S, Bladin PF (1993). The capsular warning syndrome: Pathogenesis and clinical features. Neurology.

[32] Donnan GA, Bladin PF, Berkovic SF, Longley WA, Saling MM (1991). The stroke syndrome of striatocapsular infarction. Brain.

[33] Siegler JE, Martin-Schild S (2011). Early Neurological Deterioration (END) after stroke: The END depends on the definition. Int. J. Stroke.

[34] Nam KW (2017). D-dimer as a predictor of early neurologic deterioration in cryptogenic stroke with active cancer. Eur. J. Neurol..

[35] Kim J-M (2019). Incidence and mechanism of early neurological deterioration after endovascular thrombectomy. J. Neurol..

[36] Nam K-W (2019). Serum homocysteine level is related to cerebral small vessel disease in a healthy population. Neurology.

