# Supplementary material for: Infarct growth velocity predicts early neurological outcomes in single subcortical infarction
Source: Sci Rep. 2023 Mar 18;13:4511. doi: 10.1038/s41598-023-31727-0 (PMC10024754; doi:10.1038/s41598-023-31727-0)

**Supplemental Materials**

**Supplemental Table I. Simple linear regression analysis between infarct growth velocity**^*^ **and demographic, clinical, and laboratory risk factors**

|  | *β* (95% CI) | *P* value |
| --- | --- | --- |
| Age, years | 0.002 (-0.007 to 0.011) | 0.721 |
| Sex, male | -0.139 (-0.365 to 0.086) | 0.226 |
| Time to MRI, h | -0.085 (-0.097 to -0.073) | < 0.001 |
| Hypertension | 0.128 (-0.104 to 0.360) | 0.278 |
| Diabetes | 0.308 (0.069 to 0.548) | 0.012 |
| Hyperlipidemia | 0.030 (-0.223 to 0.282) | 0.817 |
| Current smoking | -0.099 (-0.339 to 0.141) | 0.420 |
| Stroke history | -0.118 (-0.458 to 0.221) | 0.494 |
| Initial NIHSS score | 0.615 (0.450 to 0.780) | < 0.001 |
| Fasting blood sugar, mg/dL^*^ | 0.630 (0.196 to 1.064) | 0.004 |
| Hemoglobin A1c, %^*^ | 0.585 (-0.025 to 1.196) | 0.060 |
| Total cholesterol, mg/dL^*^ | -0.002 (-0.472 to 0.469) | 0.994 |
| White blood cell counts, ×10^3^/ μL^*^ | 0.562 (0.210 to 0.913) | 0.002 |
| High-sensitivity CRP, mg/dL^*^ | 0.047 (-0.038 to 0.133) | 0.280 |
| Posterior SSI | -0.931 (-1.151 to -0.710) | < 0.001 |
| Proximal SSI | 0.870 (0.636 to 1.104) | < 0.001 |
| DWI diameter, mm^*^ | 1.609 (1.461 to 1.757) | < 0.001 |
| DWI volume, mL^*^ | 0.980 (0.923 to 1.037) | < 0.001 |
| Early neurological deterioration | 0.773 (0.477 to 1.069) | < 0.001 |

MRI = magnetic resonance imaging, NIHSS = National Institutes of Health Stroke Scale, SSI = single subcortical infarction, DWI = diffusion-weighted imaging

^*^These variables were transformed into a log scale.

**Supplemental Table II. Biological interaction between proximal single subcortical infarction and infarct growth velocity for early neurological deterioration**

|  | Adjusted OR  (95% CI) | *P-*value |
| --- | --- | --- |
| Age | 1.03 [1.01-1.05] | 0.008 |
| Sex | 0.72 [0.42-1.22] | 0.225 |
| Initial NIHSS score | 1.35 [0.93-1.96] | 0.116 |
| Current smoking | 0.92 [0.48-1.77] | 0.800 |
| Proximal SSI | 4.18 [1.97-8.87] | < 0.001 |
| IGV ≥ 0.038 | 3.48 [1.81-6.70] | < 0.001 |
| Interaction [proximal SSI x IGV ≥ 0.038] | 0.31 [0.12-0.82] | 0.019 |

NIHSS = National Institutes of Health Stroke Scale, SSI = single subcortical infarction, IGV = infarct growth velocity

**Supplemental Table III. Univariate logistic regression analysis of possible predictors for early neurological deterioration according to the type of SSI**

|  | Distal type SSI  (n = 423) | | Proximal type SSI  (n = 181) | |
| --- | --- | --- | --- | --- |
|  | Crude OR  (95% CI) | *P-*value | Crude OR  (95% CI) | *P-*value |
| Age | 1.07 [1.04-1.10] | < 0.001 | 1.02 [0.99-1.05] | 0.135 |
| Sex | 0.53 [0.30-0.95] | 0.032 | 0.82 [0.42-1.61] | 0.567 |
| Time to MRI | 0.96 [0.92-1.00] | 0.031 | 0.99 [0.95-1.03] | 0.564 |
| Hypertension | 1.13 [0.62-2.04] | 0.697 | 1.39 [0.64-3.00] | 0.404 |
| Diabetes | 0.97 [0.51-1.84] | 0.926 | 1.06 [0.53-2.10] | 0.870 |
| Hyperlipidemia | 0.55 [0.26-1.17] | 0.122 | 1.04 [0.50-2.15] | 0.918 |
| Current smoking | 0.48 [0.24-0.96] | 0.038 | 1.04 [0.50-2.20] | 0.911 |
| Stroke history | 0.68 [0.26-1.79] | 0.436 | 0.91 [0.31-2.63] | 0.857 |
| Initial NIHSS score | 2.05 [1.25-3.35] | 0.004 | 1.18 [0.70-1.98] | 0.532 |
| Fasting blood sugar^*^ | 1.95 [0.65-5.84] | 0.233 | 0.78 [0.22-2.80] | 0.702 |
| Hemoglobin A1c^*^ | 2.19 [0.51-9.49] | 0.295 | 1.70 [0.29-10.03] | 0.560 |
| Total cholesterol^*^ | 2.31 [0.67-7.96] | 0.183 | 0.39 [0.08-1.91] | 0.246 |
| WBC counts^*^ | 0.87 [0.35-2.15] | 0.757 | 1.64 [0.56-4.84] | 0.367 |
| hs-CRP^*^ | 1.12 [0.91-1.38] | 0.286 | 1.09 [0.84-1.43] | 0.516 |
| Posterior circulation | 0.47 [0.23-0.94] | 0.032 | 0.96 [0.49-1.90] | 0.913 |
| DWI diameter^*^ | 3.15 [1.73-5.71] | < 0.001 | 1.16 [0.58-2.31] | 0.676 |
| DWI volume^*^ | 1.97 [1.44-2.68] | < 0.001 | 1.18 [0.89-1.57] | 0.263 |
| IGV^*^ | 1.79 [1.41-2.28] | < 0.001 | 1.09 [0.88-1.36] | 0.417 |

SSI = single subcortical infarct, MRI = magnetic resonance imaging, NIHSS = National Institutes of Health Stroke Scale, WBC = white blood cell, hs-CRP = high-sensitivity C-reactive protein, DWI = diffusion-weighted imaging, ICGV = infarct growth velocity

^*^These variables were transformed into a log scale.

**Supplemental Table IV. Comparison of characteristics between groups based on the discharge outcome^*^**

|  | **Favorable outcome**  (n = 470) | **Unfavorable outcome**  (n = 134) | ***P* value** |
| --- | --- | --- | --- |
| Early neurological deterioration | 34 (7.2) | 65 (48.5) | < 0.001 |
| Time to MRI | 10.0 [5.5-18.5] | 8.5 [4.0-16.0] | 0.118 |
| DWI volume | 0.28 [0.13-0.52] | 0.49 [0.25-0.95] | < 0.001 |
| Infarct growth velocity | 0.026 [0.013-0.061] | 0.057 [0.024-0.149] | < 0.001 |
| Initial NIHSS score | 2 [1-4] | 5 [3-6] | < 0.001 |

MRI = magnetic resonance imaging, DWI = diffusion-weighted imaging, NIHSS = National Institutes of Health Stroke Scale

**^*^**Based on the modified Rankin Scale (mRS) score at the time of discharge, the discharge outcome is divided as a favorable outcome with mRS 0-2 points and an unfavorable outcome with mRS 3-6 points.

**Supplementary Figure I. Receiver operating characteristic curve of IGV for prediction of early neurological deterioration**


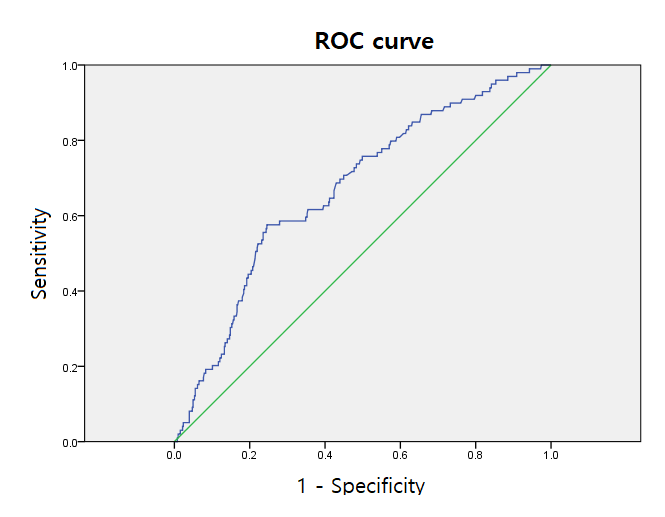

Supplement: Supplementary file 2 — Supplementary Information 2. [file 41598_2023_31727_MOESM2_ESM.docx]
